# The role of skin inflammation, barrier dysfunction, and oral tolerance in skin sensitization to gluten‐derived hydrolysates in a rat model

**DOI:** 10.1111/cod.14233

**Published:** 2022-10-22

**Authors:** Jeppe Madura Larsen, Anne‐Sofie Ravn Ballegaard, Angela Serrano Dominguez, Nanna Jordahn Kristoffersen, Natalia Zofia Maryniak, Arielle Vallee Locke, Sahar Kazemi, Michelle Epstein, Charlotte Bernhard Madsen, Katrine Lindholm Bøgh

**Affiliations:** ^1^ National Food Institute Technical University of Denmark Kongens Lyngby Denmark; ^2^ Department of Dermatology Medical University of Vienna Vienna Austria

**Keywords:** animal model, food allergy, gluten, oral tolerance, skin barrier dysfunction, skin inflammation, skin sensitization, wheat

## Abstract

**Background:**

Adverse reactions to wheat‐containing skin care products have been linked to food allergy development.

**Objectives:**

To determine the role of skin barrier dysfunction and inflammation in sensitization to gluten‐derived hydrolysates via the skin in Brown Norway rats with and without oral tolerance to wheat.

**Methods:**

Skin barrier defect was induced by mechanical disruption, and skin inflammation was induced by topical application of SLS or MC903. Unmodified, enzyme hydrolyzed, or acid hydrolyzed gluten products were applied to the skin three times per week for 5 weeks. Subsequently, rats were orally gavaged with unmodified gluten.

**Results:**

Wheat‐naïve rats were readily sensitized to gluten hydrolysates via the skin. Skin barrier defect and skin inflammation had little effect on the skin sensitization and hydrolysate‐specific IgE levels. Oral administration of unmodified gluten promoted the production of unmodified gluten‐specific IgE in rats sensitized via the skin. Sensitization through intact skin, disrupted skin barrier, or inflamed skin was unable to break tolerance to unmodified gluten in rats on a wheat‐containing diet.

**Conclusions:**

Mechanical skin barrier disruption and skin inflammation play a limited role in experimental skin sensitization to gluten‐derived hydrolysates.

## Graphical abstract

Skin barrier dysfunction and inflammation play a limited role in the cutaneous sensitization to gluten‐derived hydrolysates. Skin barrier dysfunction and inflammation do not promote breakage of oral tolerance to gluten thought cutaneous sensitization to gluten‐derived hydrolysates. Oral exposure to intact gluten drives gluten‐specific IgE production after cutaneous sensitization to gluten‐derived hydrolysates.

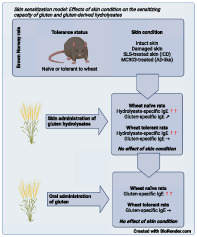

## INTRODUCTION

Food allergy is a growing health concern as the disease prevalence has increased during the past decades. Recent estimates suggest that food allergy now affects approximately 5–8% of children and 2–4% of adults.^1^ It is increasingly appreciated that the skin plays a central role in allergic sensitization to food allergens. Early studies reported that the topical use of peanut oil in infants was associated with peanut allergy development.^2^ Later studies found that environmental exposure to peanut in house dust was associated with peanut allergy in infants with atopic dermatitis (AD) or skin‐barrier defects due to loss‐of‐function mutations in the filaggrin gene.^3^, ^4^ These seminal studies indicated a role for the skin in the development of food allergy, which could be influenced by AD‐associated skin inflammation and skin barrier defects. Indeed, studies in experimental animal models of AD demonstrated that the application of food allergens on a mechanically disrupted skin barrier or AD‐like inflammatory skin lesions leads to sensitization and clinical food allergy development.^5^, ^6^, ^7^, ^8^, ^9^

A role for the skin in the development of food allergy is further supported by clinical reports of adverse reactions following the use of personal care products containing hydrolyzed wheat proteins (recently summarized by Bruusgaard‐Mouritsen et al.^10^). Wheat proteins are hydrolyzed by either proteolytic enzymes or acid to increase water solubility and to obtain products with specific physicochemical properties for use as emulsifying or foaming agents in personal care products.^11^, ^12^ A recent survey of the Danish consumer market found that wheat was the second most frequent food allergy‐associated ingredient present in 602 of the 10 067 (6%) personal care products evaluated.^10^ The study also reported that wheat is the most frequent ingredient that causes food protein‐related type‐I and type‐IV allergic reactions following the use of personal care products.^10^ Notably, a facial soap containing acid hydrolyzed gluten was responsible for more than 2000 cases of allergic reactions in Japan.^13^ A significant number of these patients became allergic to intact unmodified wheat proteins and experienced food allergy symptoms upon ingestion of food products containing unmodified wheat.^14^ These findings suggested that adverse skin reactions to the acid hydrolyzed gluten‐containing soap was able to break oral tolerance to wheat, as most patients were able to consume wheat‐containing foods before the skin‐mediated sensitization to acid hydrolyzed gluten.

We have previously characterized five different gluten‐derived products in order to identify physicochemical properties that affect sensitization via the skin in a Brown Norway (BN) rat model.^15^ We found that acid hydrolysis of gluten potentiates product sensitization via the skin, suggesting that acid hydrolyzed proteins in personal care products are potent drivers of inducing allergy through the skin. Different skin conditions may also play a role in the sensitization to gluten‐derived products; however, this matter remains largely unexplored. Soap products for skin use are a common cause of irritant contact dermatitis (ICD) to personal care products.^16^ This can be attributed to the presence of surfactants (soap compounds or detergents) with known skin irritation properties^17^ within the products. The occurrence of ICD from personal care products,^16^ and reports of adverse type‐IV skin reactions to personal care products containing food proteins^10^ highlight the need to assess the role of ICD in the development of food allergy via the skin. Here, we compare the role of mechanical skin barrier disruption, sodium lauryl sulfate (SLS)‐induced ICD, and MC903‐induced AD‐like skin inflammation in the sensitization to different gluten‐derived hydrolysates in allergy‐prone BN rats. Using rats on diets with or without wheat, we investigate the role of oral tolerance in skin sensitization to gluten hydrolysates.

## METHODS

### Gluten products

The study included five different gluten‐derived products: One intact, unmodified gluten (Un Glu), one enzyme hydrolyzed gluten (E Glu), and three acid hydrolyzed gluten products (Ac Glu 1–3), which we have previously described.^15^ Ac Glu 3 (also known as Glupearl 19S) was kindly provided by Prof. Kayoko Matsunaga, Fujita Health University School of Medicine, Aichi, Japan. The products were dissolved in sterile phosphate‐buffered saline (PBS; 137 mM NaCl, 3 mM KCl, 8 mM Na_2_HPO_4_ × 2 H_2_O, and 1 mM KH_2_PO_4_; pH 7.2). Endotoxin content was <20 EU/mg protein in all products as measured by the Pierce LAL Chromogenic Endotoxin Quantification Kit (ThermoFisher Scientific, Massachusetts) according to the manufacturer's instructions. Please refer to Ballegaard et al.^15^ for the physicochemical characterization of the products.

### Animals

BN rats were bred and maintained in‐house on a wheat‐free diet (wheat‐naïve animals) for more than 10 generations or on a wheat‐containing diet (wheat‐tolerant animals) for more than three generations at the National Food Institute, Technical University of Denmark, Denmark, as previous described.^18^, ^19^ Diet and water were given ad libitum. Rats were inspected twice daily and weighed once a week. Ethical approval was provided by the Danish Animal Experiments Inspectorate (authorization no. 2015‐15‐0201‐00553‐C1). Experiments were overseen by the National Food Institute's in‐house Animal Welfare Committee for animal care and use.

### Pilot study

SLS‐induced skin inflammation is a well‐known model of ICD,^20^ and the vitamin D analogue MC903 has been shown to induce AD‐like skin inflammation via keratinocyte‐derived TSLP production in mice.^21^ The induction of skin inflammation by SLS and MC903 in BN rats was confirmed in a pilot study. SLS (7% [w/v], 100 μl; Sigma‐Aldrich, St. Louis, Missouri) or MC903 (100 nmol/ml, 100 μl; Sigma‐Aldrich) was applied to shaven abdominal skin (on an area of approximately 2 × 2 cm) of BN rats (males, age 5–6 weeks, *n* = 2/group) 5 times/week for 2 weeks (Day 0–4 and 7–11). SLS‐ and MC903‐treated skin was compared with untreated intact skin and skin treated with sandpaper grit 400 once a week for 2 weeks (Days 0 and 7) giving rise to damaged skin with impaired skin barrier.^9^ The induction of skin damage and inflammation was assessed clinically and evaluated by histology after sacrifice.

### Experimental design

Skin‐mediated sensitization to five gluten‐derived products was evaluated across four different skin conditions: Intact skin, mechanically disrupted skin barrier (damaged skin), and skin inflammation induced by SLS or MC903. Experiments were performed in sets to have enough animals within the age range of 5–8 weeks (intact, damaged, SLS, and MC903; *n* = 6–8/group with equal numbers of males and females). We previously reported the development of a mechanically disrupted skin barrier model (damaged skin) in BN rats, where the abdominal skin was lightly damaged by sandpaper scratching once a week for up to 5 weeks giving rise to increased transepidermal water loss (TEWL) and keratinization, but not skin inflammation as evaluated by histology.^9^ Here, abdominal skin was shaved using an electric razor once a week (Days 0, 7, 14, 21, 28, and 35) or more often if needed. Intact skin was only shaven, whereas damaged skin was induced by light scratching using sandpaper (grit 400) once a week for 5 weeks (Days 7, 14, 21, 28, and 35) on the shaven abdominal skin. Skin inflammation was induced by topical application of SLS (7% [w/v], 100 μl) or MC903 (100 nmol/ml, 100 μl) 5 times/week for 6 weeks (Days 0–4, 7–11, 14–18, 21–25, 28–32, and 35–39) on shaven abdominal skin. Gluten‐derived products (5 mg/ml in PBS, 100 μl) or PBS alone were applied to the different skin conditions 3 times/week for 5 weeks (Days 7–9, 14–16, 21–23, 28–30, and 35–37). To avoid oral exposure, the product‐dosed skin was covered with an elastic gauze bandage for 1 h and subsequently rinsed with water. In order not to be washed away, SLS or MC903 applications were performed after skin application of gluten‐derived products. Following the skin application regimen, rats were postimmunized by oral gavages containing Un Glu (50 mg in 1 ml PBS) to simulate ingestion of unmodified gluten after skin‐mediated sensitization (Days 42 and 49). BN rats were sacrificed by exsanguination using carbon dioxide as anaesthesia 1 week after the last postimmunization on Day 57. Blood samples were collected before and after oral postimmunizations (Days 42 and 57).

### Skin barrier function

TEWL was compared between the rats with different skin conditions on the day of the first application of gluten‐derived products to the skin (Day 7) by calculating the change in TEWL compared with baseline for each skin condition (Intact skin, SLS, and MC903: Day 0 vs. 7. Damaged skin: Before vs. after sandpaper treatment on Day 7) using a Tewameter TM 300 (Courage+Khazaka Electronic GmbH, Cologne, Germany).

### Ear swelling test

The capacity of Un Glu to elicit a clinical allergic response was evaluated by an ear swelling test (EST) 1 day before animal sacrifice (Day 56), as previously described.^22^, ^23^ Briefly, Un Glu was injected intradermally in the ear (3 μg/dose in 20 μl PBS), and ear thickness was measured before and 1 h after injection using a Digital Micrometre (range 0–25 mm, resolution 0.001 mm, accuracy ±0.002 mm; RS PRO, Corby, United Kingdom).

### Histology

Induction of skin damage and inflammation was evaluated by histology of intact skin, damage skin, and skin treated with SLS or MC903 in the pilot study. Abdominal skin tissue was fixed in 10% neutral buffered formalin (Hounisen, Skanderborg, Denmark) for 24 h. Tissues were dehydrated through increasing ethanol series (70–99%), rendered permeable with xylene, and embedded in paraffin. Tissues were cut in 5–6 μm sections using a microtome Microm HM 360 (Thermo Scientific, MA). All sections were stained with Haematoxylin–Eosin (HE; Ampliqon, Odense, Denmark). Slides were analysed using a Leica DM2500 LED microscope with the Leica Application Suite X version 5 software package (Leica Microsystems CMS GmbH Mannheim, Germany).

### Product‐specific IgG1 by Indirect ELISA

Gluten‐derived product‐specific IgG1 was analysed by indirect ELISA as previously described.^9^, ^15^ Briefly, ELISA plates were coated with the respective gluten‐derived product (Un Glu, E Glu, Ac Glu 1, 2, 3). Two‐fold serial dilutions of serum samples were incubated on plates to obtain antibody Log_2_ titre values. IgG1 antibodies were detected using horseradish peroxidase (HRP)‐labelled mouse anti‐rat IgG1 (3060‐05, Southern Biotech, Birmingham, Alabama) with 3,3′,5,5′‐tetramethylbenzidine (TMB)‐one (4380A, Kementec Diagnostics, Taastrup, Denmark) as substrate.

### Product‐specific IgE by capture ELISA

Gluten‐derived product‐specific IgE was analysed by IgE‐capture ELISA as previously described,^9^, ^15^ with the exception, that serum samples were diluted in 3% (v/v) rabbit serum (Almeco, Esbjerg, Denmark; Un Glu) or 3% (w/v) skimmed milk powder (Sigma‐Aldrich; E Glu, and Ac Glu 1–3). Briefly, ELISA plates were coated with mouse anti‐rat IgE antibody (HDMAB‐123 HydriDomus, Nottingham, United Kingdom). Two‐fold serial dilutions of serum samples were incubated on plates to obtain antibody Log_2_ titre values. Specific IgE antibodies were detected using digoxigenin (DIG)‐coupled gluten‐derived product and HRP‐labelled sheep anti‐DIG antibody (11 633 716 001, Roche Diagnostics GmbH, Mannheim, Germany) with TMB‐one as substrate.

### Statistical analyses

Product‐specific IgG1 and IgE levels were compared with the PBS control groups using the Mann–Whitney test. Differences in Un Glu‐specific IgG1 and IgE levels, and EST responses to Un Glu were compared with the PBS control groups using the Kruskal‐Wallis test, and correction for multiple comparisons was performed by controlling the False Discovery Rate (FDR) using the method of Benjamini, Krieger, and Yekutieli. Un Glu‐specific IgE and IgG1 levels before versus after oral Un Glu exposure were evaluated using the Wilcoxon test. The effects of gluten‐derived products and skin conditions were additionally evaluated using 2‐way ANOVA including the following groups: E Glu and Ac Glu 1–3 groups (product‐specific IgE data), or Un Glu, E Glu, and Ac Glu 1–3 groups (Un Glu‐specific IgE data). ANOVA post‐hoc multiple comparisons were performed by controlling the FDR using the method of Benjamini, Krieger, and Yekutieli. The levels of statistically significant differences are indicated using asterisks (*p < 0.05; **p < 0.01; and ***p < 0.001). Data analysis and visualization were performed with GraphPad Prism version 9.3 (San Diego, CA).

## RESULTS

### SLS and MC903 induce skin inflammation in BN rats

**FIGURE 1:**
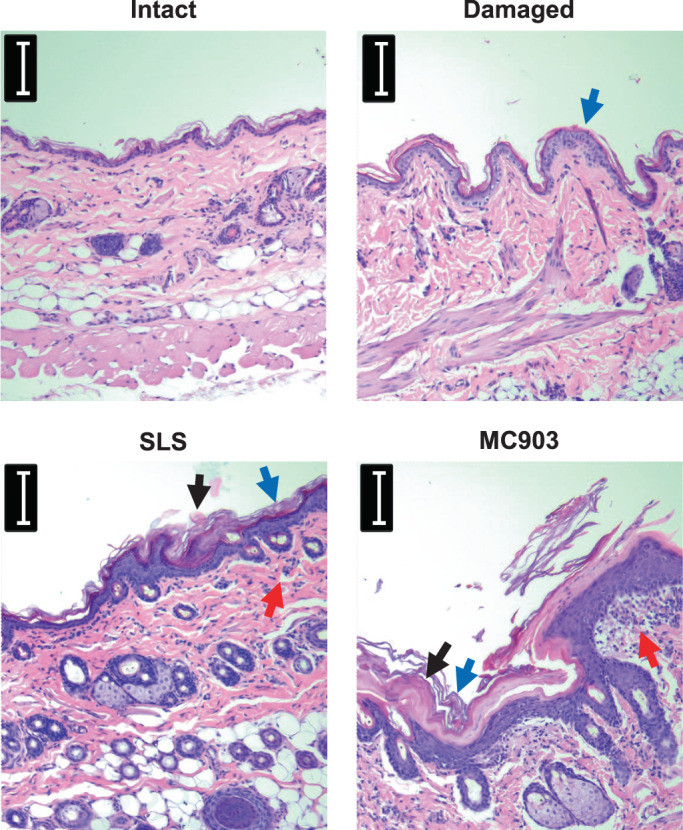
Histology of intact skin, barrier disrupted skin, and SLS‐ or MC903‐treated skin from the pilot study in Brown Norway rats. Damaged skin induced by light scratching with sandpaper once a week for 2 weeks (Days 0 and 7). Skin inflammation was induced by SLS or MC903 application to skin of Brown Norway rats 5 times/week for 2 weeks (Day 0–4 and 7–11). Induction of inflammation was evaluated by histology using haematoxylin and eosin (HE)‐stained skin sections collected after sacrifice. The bar shows the length of 50 μm. Examples of fibrin deposition, epidermal thickening with enhanced keratinization, and infiltration of mononuclear cells are indicated using black, blue, and red arrows, respectively.

Models of impaired skin barrier function^9^ and skin inflammation were established to study sensitization to gluten‐derived products via the skin in BN rats with or without oral tolerance to wheat. Topical application of SLS and MC903 has previously been shown to induce ICD^20^ and AD‐like skin inflammation,^21^ respectively. A pilot study in BN rats was performed to evaluate the induction of clinical skin inflammation in response to SLS and MC903 in comparison to intact and damaged skin. We observed that SLS‐treated rats presented with skin lesions characterized by dryness and flaky skin. Similarly, MC903‐treated rats developed skin lesions characterized by dryness and flaky skin with patches of erythema. Damaged skin showed no clinical signs of inflammation. Histological evaluation of HE‐stained skin sections revealed that SLS treatment induced epidermal thickening with enhanced keratinization, fibrin deposition, and moderate dermal infiltration of mononuclear cells (Figure 1). Sections of MC903‐treated skin revealed epidermal thickening with enhanced keratinization, fibrin deposition, and infiltration of mononuclear cells close to the epidermal layer. Sections of mechanically damaged skin revealed slight epidermal thickening with enhanced keratinization and with no infiltration of mononuclear cells.

### Skin barrier dysfunction and inflammation have limited effect on skin sensitization to gluten‐derived products in wheat‐naïve rats

**FIGURE 2:**
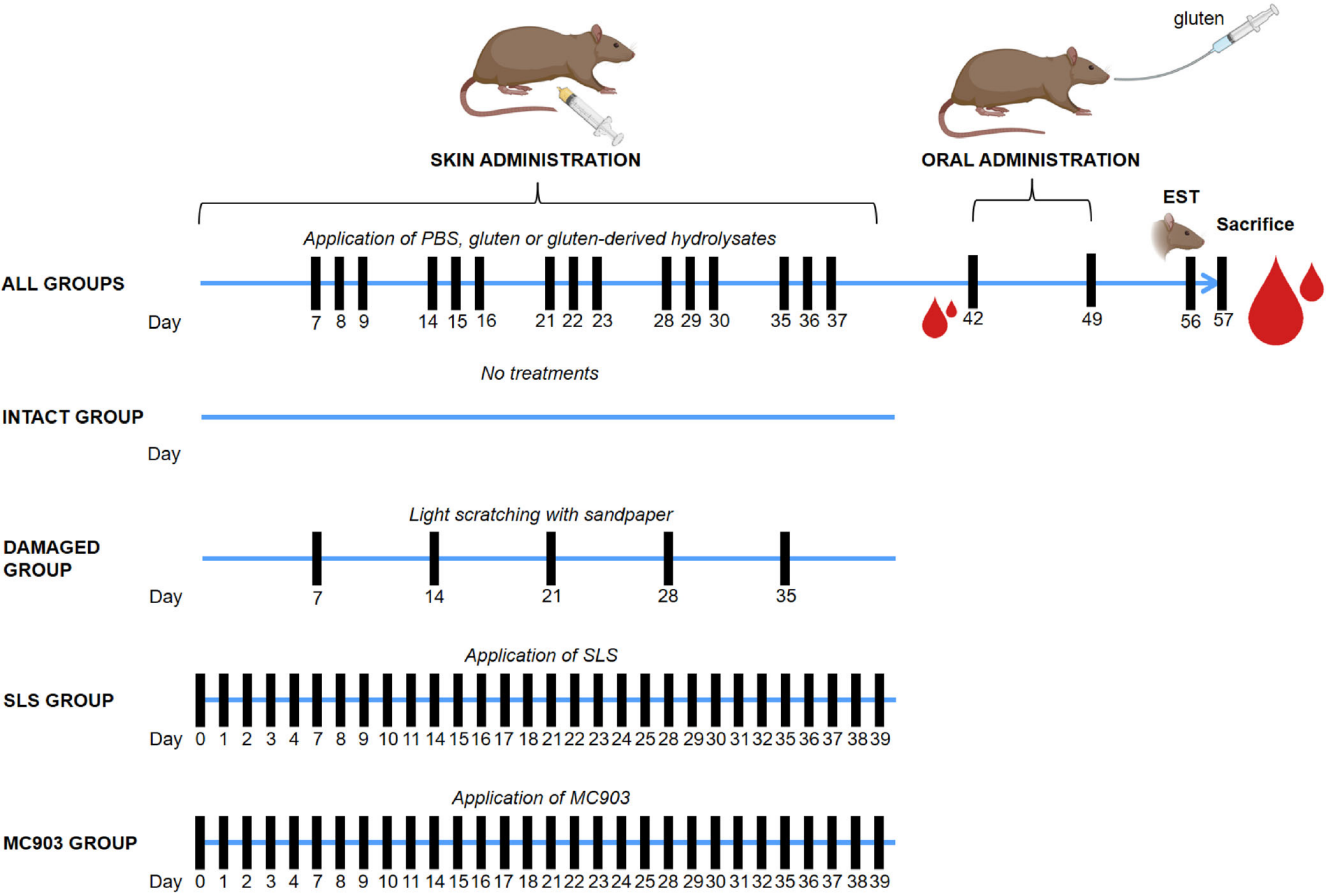
Animal experimental design. Barrier disrupted skin (damaged) was induced by slightly scratching using sandpaper once a week for 5 weeks (Days 7, 14, 21, 28, and 35) on shaven skin in Brown Norway rats. Skin inflammation was induced by topical application of sodium lauryl sulphate (SLS) or the vitamin D analogue MC903 5 times/week for 6 weeks (Days 0–4, 7–11, 14–18, 21–25, 28–32, and 35–39) on shaven skin. Intact skin was only shaven. Gluten‐derived products (unmodified gluten (Un Glu), enzyme hydrolyzed gluten (E Glu), or acid hydrolyzed gluten (Ac Glu 1–3) or PBS alone were applied to the rats with different skin conditions 3 times/week for 5 weeks (Days 7–9, 14–16, 21–23, 28–30, and 35–37). Oral gavages with Un Glu were administrated on Days 42 and 49. Blood samples were collected before and after the oral gavages (Days 42 and 57). An ear swelling test (EST) to Un Glu was performed on Day 56, and rats were sacrificed Day 57. Pictures were provided by BioRender.com.

**FIGURE 3:**
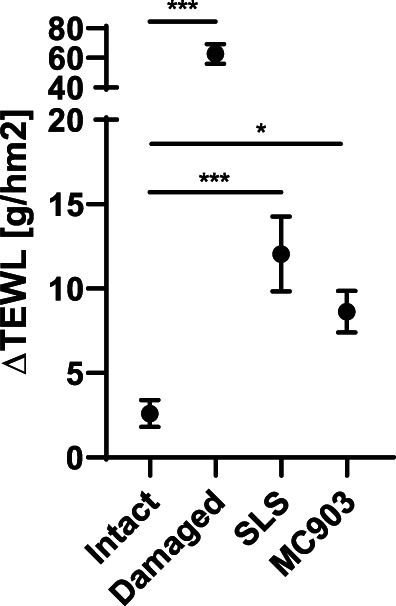
Transepidermal water loss across intact skin, barrier disrupted skin, or inflamed skin induced by SLS or MC903 in wheat‐naïve Brown Norway rats. Change in transepidermal water loss (TEWL) at the time of the first skin application of gluten‐derived products compared with baseline (Day 0 vs. 7 [intact, and SLS‐ or MC903‐treated skin], and before vs. after sandpaper treatment [barrier disrupted defect, damaged]). Data show mean (±SEM) change in TEWL (*n* = 12–48 per group). The level of statistically significant differences between indicated groups are shown using asterisks: *p < 0.05; **p < 0.01; ***p < 0.001.

**FIGURE 4:**
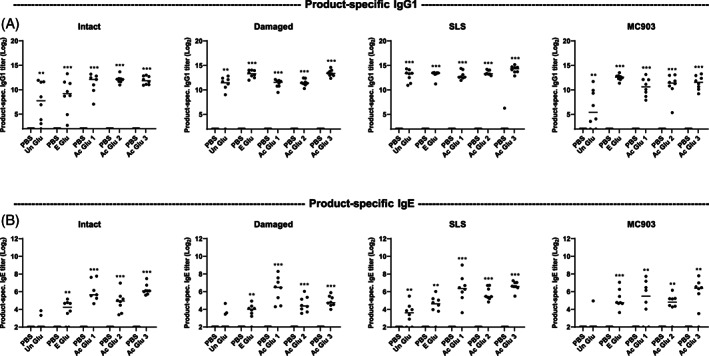
Product‐specific IgG1 and IgE levels following application of gluten‐derived products to intact skin, barrier disrupted skin, or inflamed skin induced by SLS or MC903 in wheat‐naïve rats. Product‐specific (A) IgG1 and (B) IgE levels following 5 weeks of application (Day 42) of unmodified gluten (Un Glu), enzyme hydrolyzed gluten (E Glu), or acid hydrolyzed gluten (Ac Glu 1–3) products to intact skin, barrier disrupted skin (damaged), or inflamed skin induced by SLS or MC903 in wheat‐naïve Brown Norway rats. Each symbol represents a single rat and horizontal lines indicate median values (*n* = 7–8 per group). The level of statistically significant differences between indicated groups are shown using asterisks: *p < 0.05; **p < 0.01; ***p < 0.001.

Skin‐mediated sensitization to gluten‐derived products was evaluated across the established skin conditions: Intact skin, damaged skin, and skin inflammation induced by SLS or MC903 (Figure 2). Five different gluten products were included in the study: One unmodified intact gluten (Un Glu), one enzyme hydrolyzed gluten product (E Glu), and three different acid hydrolyzed gluten products (Ac Glu 1–3). The capacity of the gluten‐derived products to induce product‐specific IgG1 and IgE in serum after topical application in the context of the established skin conditions were evaluated in wheat‐naïve BN rats. Skin barrier function was initially evaluated by TEWL at the time of the first application of the gluten‐derived product. Damaged, SLS‐treated, and MC903‐treated skin were characterized by increased TEWL compared with intact skin, confirming barrier dysfunction in the damaged, SLS and MC903 skin groups (Figure 3). All gluten products were found to induce product‐specific IgG1 after 5 weeks of topical application, when compared with control groups with PBS applied to the skin (Figure 4A). This finding demonstrates that the products penetrate the skin, that they are recognized by the cutaneous immune system, and that specific immune responses develop. The hydrolyzed gluten products were all found to induce sensitization via the skin, but the levels of product‐specific IgE seemed unaffected by skin damage or inflammation, as the pattern of IgE levels were comparable between the different skin conditions (Figure 4B). Indeed, a direct comparison of the effect of skin condition on IgE levels for each of the gluten products supported a limited effect of skin barrier dysfunction and inflammation (Figure S1). These observations were further supported by a 2‐way ANOVA analysis, which found that the product‐specific IgE levels were mainly influenced by the gluten products applied to the skin (21.7% of the variation, *p* < 0.0001) and less by the skin condition (5.4% of the variation, *p* = 0.0287). The post‐hoc analysis revealed that the only skin condition‐associated difference in product‐specific IgE levels could be ascribed to SLS‐treated skin promoting IgE levels compared with damaged skin (0.9205 mean difference, *p* = 0.0037). It should be noted that the experiment was divided into different sets according to the different skin conditions. The product‐specific IgE levels were highly influenced by the specific gluten product applied to the skin, and aggregation of the results indicated that the Ac Glu 1–3 products induced higher IgE levels compared with E Glu, which was higher than Un Glu (Figure S2).

### Oral exposure to unmodified gluten promotes IgE production against unmodified gluten in wheat‐naïve rats sensitized to hydrolyzed gluten‐derived products via the skin

**FIGURE 5:**
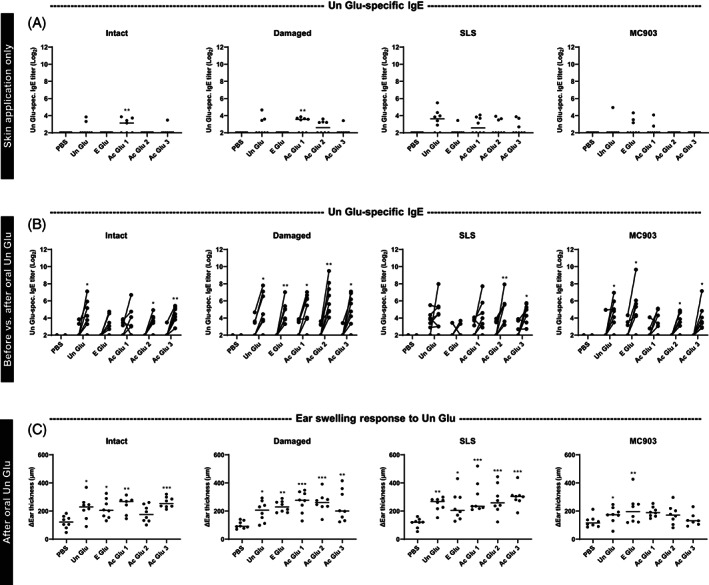
Cross‐reactivity and clinical reactivity to unmodified gluten following application of gluten‐derived products to intact skin, barrier disrupted skin, or inflamed skin induced by SLS or MC903 in wheat‐naïve rats. Unmodified gluten (Un Glu)‐specific IgE levels (A) before (Day 42), and (B) before (Day 42) versus after (Day 57) two oral gavages of Un Glu following 5 weeks application of Un Glu, enzyme hydrolyzed gluten (E Glu), or acid hydrolyzed gluten (Ac Glu 1–3) products to intact skin, barrier disrupted skin (damaged), or inflamed skin induced by SLS or MC903 in wheat‐naïve Brown Norway rats. (C) Ear swelling in response (Day 56) to intradermal Un Glu injections following the two oral gavages of Un Glu. Each symbol represents a single rat and horizontal lines indicate median values (*n* = 7–8 per group). The level of statistically significant differences between before and after oral postimmunization with Un Glu (B) and between indicated groups (C) are shown using asterisk: *p < 0.05; **p < 0.01; ***p < 0.001.

Cross‐reactivity to Un Glu was evaluated after exposure to gluten‐derived products via the skin in wheat‐naïve BN rats. Overall, the application of hydrolyzed gluten‐derived products induced limited IgE cross‐reactivity to Un Glu (Figure 5A). Forty‐three of 159 rats (27%) had detectable Un Glu‐specific IgE. Still, Un Glu‐specific IgG1 was detectable in almost all rats (154 of 159, 97%, see Figure S3A) and at levels comparable to the product‐specific IgG1 levels (Figure 4A). These findings indicate that the rats were exposed to Un Glu‐associated epitopes via skin application of the hydrolyzed products.

After skin application of gluten‐derived products, the rats were subsequently gavaged twice with Un Glu to simulate eating wheat after exposure to gluten‐derived products on the skin. Interestingly, oral exposure to Un Glu promoted Un Glu‐specific IgE production in animals exposed to gluten‐derived products, but not PBS, on the skin (Figure 5B). At this point, 129 of 158 rats (81.6%) had detectable Un Glu‐specific IgE. In contrast, Un Glu‐specific IgG1 levels were less affected by oral exposure to Un Glu (Figure S3B). Two‐way ANOVA analysis found that Un Glu‐specific IgE levels after oral exposure were not affected by gluten‐derived products applied to the skin (4.2% of the variation, *p* = 0.1112), and that the skin condition contributed to 9.4% of the variation (*p* = 0.0010). The post‐hoc analysis found that the effect of the skin condition could be ascribed to higher IgE levels mediated by damaged skin compared with intact (1.367 mean difference, *p* = 0.0002), SLS‐treated (1.070 mean difference, *p* = 0.0031), and MC903‐treated (1.107 mean difference, *p* = 0.0024) skin. It should be noted that the experiment was divided into different sets according to the different skin conditions.

An EST was performed to evaluate the clinical reactivity to Un Glu after skin application of gluten‐derived products and subsequent oral administration of Un Glu (Figure 5C). Overall, the increase in ear thickness in response to Un Glu correlated with Un Glu‐specific IgE levels in serum (*r*_*s*_ = 0.44, *p* < 0.0001, Figure S4). The results demonstrate the development of functional Un Glu‐specific IgE after exposure to the gluten‐derived product on the skin and subsequent oral administration of Un Glu.

### Skin barrier dysfunction and inflammation have limited effect on skin sensitization to gluten‐derived products in wheat‐tolerant rats

**FIGURE 6:**
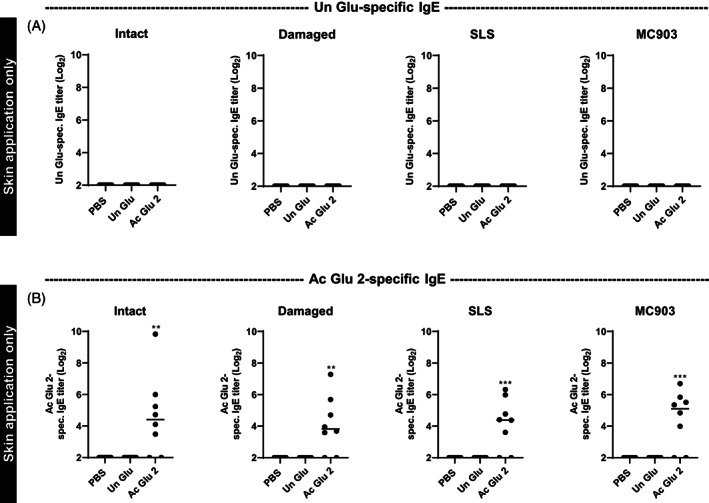
Product‐specific IgG1 and IgE levels following application of gluten‐derived products to intact skin, barrier disrupted skin, or inflamed skin induced by SLS or MC903 in wheat‐tolerant rats. Product‐specific (A) IgG1 and (B) IgE levels following 5 weeks of application (Day 42) of PBS, unmodified gluten (Un Glu), or acid hydrolyzed gluten (Ac Glu 2) products to intact skin, barrier disrupted skin (damaged), or inflamed skin induced by SLS or MC903 in wheat‐tolerant Brown Norway rats. Each symbol represents a single rat and horizontal lines indicate median values (*n* = 6–8 per group). The level of statistically significant differences between control PBS group and treatment groups are shown using asterisks: *p < 0.05; **p < 0.01; ***p < 0.001.

Gluten‐derived hydrolysates were found to readily sensitize via the skin in wheat‐naïve rats independent of skin damage and inflammation. The role of oral tolerance was investigated by performing the experiments in rats on a wheat‐containing diet using selected gluten‐derived products (Un Glu and Ac Glu 2). Skin application of Un Glu or Ac Glu 2 was unable to break established oral tolerance to Un Glu, as no rats became sensitized to Un Glu (Figure 6A). Only Ac Glu 2 was able to induce sensitization to Ac Glu 2 (Figure 6B). The level of Ac Glu 2‐specific IgE was not affected by the skin condition. Subsequent oral exposure to Un Glu was not able to break the tolerance to Un Glu in tolerant rats exposed to Un Glu or Ac Glu 2 on the skin (data not shown).

## DISCUSSION

The use of skin care products containing hydrolyzed wheat‐derived ingredients has been linked to adverse skin reactions and the development of food allergies.^10^ Here, we investigated skin‐mediated sensitization to gluten‐derived products in the context of intact skin, a mechanically disrupted skin barrier, and skin inflammation induced by SLS and MC903. Topical application of SLS and MC903 are widely used models of ICD and AD, respectively. Here, we found that SLS and MC903 application in BN rats induced skin inflammation, characterized by clinical inflammation, increased TEWL, and dermal infiltration of mononuclear cells. This is in contrast to the damaged skin condition, which showed no clinical signs of inflammation and no dermal infiltration of mononuclear cells but exhibited increased TEWL. These findings support the absence of inflammation in the damaged skin condition, which is in line with our previous study.^9^ We propose that the damaged skin condition could be consider a model of the skin barrier defect associated with filaggrin loss‐of‐function mutations leading to increased TEWL without the inflammation seen in active AD.

The mechanically disrupted skin barrier and skin inflammation induced by SLS or MC903 were found to have little effect on skin‐mediated sensitization to the gluten‐derived products in rats without oral tolerance to wheat. A previous study of skin‐sensitization to unmodified gluten and acid hydrolyzed gluten in mice indicated that co‐application with SLS did not promote sensitization compared with intact skin,^24^ which is in line with our current findings. Yet, studies of MC903‐induced AD‐like skin inflammation in mice found that skin inflammation was needed to induce sensitization to the egg allergen ovalbumin (OVA) via the skin.^7^, ^25^ Application of OVA on intact skin did not give rise to the development of OVA‐specific IgE and clinical food allergy. Another study using Flaky Tail (ft/ft) mice with AD‐associated functional mutations in the filaggrin gene found that the mice with a dysfunctional skin barrier could be sensitized by topical application of OVA, whereas wild type (WT) mice could not.^26^ These studies suggest that a skin barrier defect and/or inflammation is needed for food allergy development via cutaneous sensitization in the mouse. However, a study comparing sensitization to several different food allergens via the skin in the absence of adjuvant demonstrated that peanut extract, cashew extract, and the major peanut allergen Ara h 2 were able to sensitize via the skin, whereas soy extract, green bean extract, and the major cow's milk allergen Bos d 4 were only able to sensitize in the presence of adjuvant.^6^ These findings highlight that both skin condition and allergen potency determine the outcome of sensitization to food allergens via the skin. Some allergenic foods may require skin barrier dysfunction and/or skin inflammation to induce sensitization. This may lead to the interpretation that wheat is a potent skin sensitizing allergen, as skin barrier defect or inflammation did not promote sensitization in our model. Conversely, the BN rat is a known high‐IgE responder strain with a predisposition to atopy.^27^ Thus, skin barrier defects or inflammation may play a minor role in the sensitization process due to this predisposition. Nevertheless, we found that the acid hydrolyzed gluten products exhibited the greatest sensitizing capacity compared with Un Glu and E Glu, which is in line with previous studies of skin‐mediated and intraperitoneal sensitization.^9^, ^15^, ^19^, ^24^, ^28^, ^29^, ^30^ Thus, skin sensitization in BN rats may be a potential tool for evaluating the sensitizing potential of food proteins in food allergy risk assessment.

Skin‐mediated sensitization to the gluten‐derived hydrolysates induced limited IgE cross‐reactivity to Un Glu in wheat‐naïve rats before oral exposure to Un Glu. This is surprising, because we previously found, that all the hydrolyzed products retained the native antigenic epitopes present in Un Glu.^15^ Indeed, Un Glu‐specific IgG1 levels were comparable to the product‐specific IgG1 levels, indicating that the immune system is exposed to Un Glu epitopes. However, hydrolyzed gluten‐derived product‐specific IgE could be detected at higher levels compared with Un Glu‐specific IgE levels, which leads us to speculate that it is the modified parts of gluten that drive the intrinsic allergenicity of the hydrolyzed products.

In the design of the present study, we sought to ensure that the only route of sensitization was the skin. To this end, the skin was covered following the application of the gluten‐derived product and washed after 1 h. This was done to minimize the potential oral and respiratory exposure caused by the grooming activities of the rats allowing for controlled oral exposure. Oral administration of Un Glu was performed to simulate ingestion of wheat after sensitization to gluten‐derived hydrolysates via the skin. Interestingly, oral gavage with Un Glu promoted the production of Un Glu‐specific IgE in animals that were exposed to gluten‐derived products, but not PBS, on the skin. This finding suggests that oral exposure after skin sensitization promotes allergic responses. This phenomenon warrants further investigation, including the study of other allergens, the amount of allergen required to sensitize via the skin, the kinetics of IgE levels following oral exposure, and the underlying mechanisms. Of note, we have previously observed at clear dose–response in sensitization to gluten‐derived products (5–500 μg), and our results indicated that a 100‐fold reduction in the dose (5 μg) of E Glu could lead to sensitization when applied to the skin.^15^

The application of Un Glu or Ac Glu 2 to intact skin, mechanically barrier disrupted skin, and inflamed skin induced by SLS or MC903 was unable to break oral tolerance to Un Glu in wheat‐tolerant rats. This finding is surprising because adverse skin reactions to an acid hydrolyzed gluten‐containing facial soap caused the break of oral tolerance to wheat in several patients.^13^ Yet, our findings align with the dual‐allergen exposure hypothesis, which proposes that skin exposure to allergens leads to sensitization if tolerance through oral exposure had not been established first.^31^ The reported break of oral tolerance in humans following skin sensitization to gluten products^13^ may be due to incomplete oral tolerance via the genetic predisposition in these individuals.^14^ We propose, that incomplete oral tolerance can be viewed as a situation, where a protein has a certain number of possible epitopes in a given host, but only some of them are controlled by tolerance mechanisms (e.g., by deletion of protein‐specific B cells or T cells, and/or induction of protein‐specific regulatory cells) whereby the tolerance for that protein is incomplete. We speculate that under certain conditions, “uncontrolled” epitopes in a host with incomplete tolerance could lead to allergic sensitization to these epitopes, and that more uncontrolled epitopes in a host leads to increased risk for break of tolerance.

Recent mechanistic studies highlight that under homeostatic conditions the skin comprises a unique noninflammatory microenvironment that fosters allergic sensitization.^32^ These findings support the notion that sensitization is the “default” immune response in the skin. Skin barrier defect and skin inflammation may exacerbate the risk of sensitization by increasing the cutaneous permeability to proteins. Thus, appropriate maintenance of skin barrier function may be important in the prevention of allergic disease. Our findings further support the view that oral tolerance should be established before environmental/skin exposure to allergenic foods. Thus, new strategies and regulations for the prevention of environmental exposure to food proteins in cosmetics and other nonfood consumer products may be important for the prevention of food allergy.

## AUTHOR CONTRIBUTIONS

Author contributions are reported in accordance to the Contributor Roles Taxonomy (CRediT). Conceptualization: Katrine Lindholm Bøgh, Charlotte Bernhard Madsen, and Jeppe Madura Larsen. Funding acquisition: Katrine Lindholm Bøgh and SK. Investigation and data curation: Jeppe Madura Larsen, Anne‐Sofie Ravn Ballegaard, Angela Serrano Dominguez, Nanna Jordahn Kristoffersen, Natalia Zofia Maryniak, Arielle Vallee Locke, Sahar Kazemi, and Katrine Lindholm Bøgh. Methodology: Katrine Lindholm Bøgh, Anne‐Sofie Ravn Ballegaard, Jeppe Madura Larsen, and Charlotte Bernhard Madsen. Visualization: Jeppe Madura Larsen and Katrine Lindholm Bøgh. Formal Analysis: Jeppe Madura Larsen. Supervision: Katrine Lindholm Bøgh and Jeppe Madura Larsen. Writing—original draft: Jeppe Madura Larsen. Writing—review & editing: Katrine Lindholm Bøgh. All authors made substantial intellectual contributions to the study, reviewed the manuscript critically, and approved the final version of the manuscript.

## CONFLICT OF INTEREST

The authors declare no conflict of interest. The funding agencies and the providers of gluten‐derived products played no role in study design, data acquisition, data analysis, interpretation, manuscript preparation, or the decision to publish.

## Supporting information


**FIGURE S1** Product‐specific IgG1 and IgE levels following application of gluten‐derived products to intact skin, barrier disrupted skin, or inflamed skin induced by SLS or MC903 in wheat‐naïve rats. Product‐specific (A) IgG1 and (B) IgE levels following 5 weeks of application (Day 42) of unmodified gluten (Un Glu), enzyme hydrolyzed gluten (E Glu), or acid hydrolyzed gluten (Ac Glu 1‐3) products to intact skin, barrier disrupted skin (damaged), or inflamed skin induced by SLS or MC903 in wheat‐naïve Brown Norway rats. Each symbol represents a single rat and horizontal lines indicate median values (*n* = 7–8 per group). The level of statistically significant differences between indicated groups are shown using asterisks: *p < 0.05; **p < 0.01; ***p < 0.001.
**FIGURE S2** Product‐specific IgG1 and IgE levels following application of gluten‐derived products to intact skin, barrier disrupted skin, or inflamed skin induced by SLS or MC903 in wheat‐naïve rats. Product‐specific (A) IgG1 and (B) IgE levels following 5 weeks of application (Day 42) of unmodified gluten (Un Glu), enzyme hydrolyzed gluten (E Glu), or acid hydrolyzed gluten (Ac Glu 1‐3) products to intact skin, barrier disrupted skin (damaged), or inflamed skin induced by SLS or MC903 in wheat‐naïve Brown Norway rats. Data shown are pool from the four different skin conditions. Each symbol represents a single rat and horizontal lines indicate median values (*n* = 31–32 per group). Statistically significant differences between groups are indicated as: *p < 0.05; **p < 0.01; ***p < 0.001.
**FIGURE S3** Cross‐reactivity to unmodified gluten following application of gluten‐derived products to intact skin, barrier disrupted skin, or inflamed skin induced by SLS or MC903 in wheat‐naïve rats. Unmodified gluten (Un Glu)‐specific IgG1 levels (A) before (Day 42) and (B) before (Day 42) versus after (Day 57) two oral gavages of Un Glu following 5 weeks of application of Un Glu, enzyme hydrolyzed gluten (E Glu), or acid hydrolyzed gluten (Ac Glu 1‐3) products to intact skin, barrier disrupted skin (damaged), or inflamed skin induced by SLS or MC903 in wheat‐naïve Brown Norway rats. Each symbol represents a single rat and horizontal lines indicate median values (*n* = 7–8 per group). Statistically significant differences between groups are indicated as: *p < 0.05; **p < 0.01; ***p < 0.001.
**FIGURE S4** Correlation between unmodified gluten‐specific IgE levels and Ear Swelling Test responses to unmodified gluten after skin application of gluten‐derived products and subsequent oral administration of unmodified gluten in wheat‐naïve Brown Norway rats. Unmodified gluten (Un Glu)‐specific IgE levels in serum after two oral gavages of Un Glu following 5 weeks of application of Un Glu, enzyme hydrolyzed gluten (E Glu), or acid hydrolyzed gluten (Ac Glu 1‐3) products to intact skin, barrier disrupted skin (damaged), or inflamed skin induced by SLS or MC903 in wheat‐naïve Brown Norway rats (Day 57). Ear Swelling Test (EST) response to intradermal Un Glu injections at Day 56. Each symbol represents a single rat (*n* = 190). Correlation was analyzed by Spearman's rank correlation test. Line shows linear regression with 95% confidence bands.

## Data Availability

The data that support the findings of this study are available from the corresponding author upon reasonable request.
