# Supplementary material for: The role of skin inflammation, barrier dysfunction, and oral tolerance in skin sensitization to gluten‐derived hydrolysates in a rat model
Source: Contact Dermatitis. 2022 Oct 22;88(2):109–19. doi: 10.1111/cod.14233 (PMC10091953; doi:10.1111/cod.14233)
Supplement: Supplementary file 1 — FIGURE S1 Product‐specific IgG1 and IgE levels following application of gluten‐derived products to intact skin, barrier disrupted skin, or inflamed skin induced by SLS or MC903 in wheat‐naïve rats. Product‐specific (A) IgG1 and (B) IgE levels following 5 weeks of application (Day 42) of unmodified gluten (Un Glu), enzyme hydrolyzed gluten (E Glu), or acid hydrolyzed gluten (Ac Glu 1‐3) products to intact skin, barrier disrupted skin (damaged), or inflamed skin induced by SLS or MC903 in wheat‐naïve Brown Norway rats. Each symbol represents a single rat and horizontal lines indicate median values (n = 7–8 per group). The level of statistically significant differences between indicated groups are shown using asterisks: *p < 0.05; **p < 0.01; ***p < 0.001. FIGURE S2 Product‐specific IgG1 and IgE levels following application of gluten‐derived products to intact skin, barrier disrupted skin, or inflamed skin induced by SLS or MC903 in wheat‐naïve rats. Product‐specific (A) IgG1 and (B) IgE levels following 5 weeks of application (Day 42) of unmodified gluten (Un Glu), enzyme hydrolyzed gluten (E Glu), or acid hydrolyzed gluten (Ac Glu 1‐3) products to intact skin, barrier disrupted skin (damaged), or inflamed skin induced by SLS or MC903 in wheat‐naïve Brown Norway rats. Data shown are pool from the four different skin conditions. Each symbol represents a single rat and horizontal lines indicate median values (n = 31–32 per group). Statistically significant differences between groups are indicated as: *p < 0.05; **p < 0.01; ***p < 0.001. FIGURE S3 Cross‐reactivity to unmodified gluten following application of gluten‐derived products to intact skin, barrier disrupted skin, or inflamed skin induced by SLS or MC903 in wheat‐naïve rats. Unmodified gluten (Un Glu)‐specific IgG1 levels (A) before (Day 42) and (B) before (Day 42) versus after (Day 57) two oral gavages of Un Glu following 5 weeks of application of Un Glu, enzyme hydrolyzed gluten (E Glu), or acid hydrolyzed glut [file COD-88-109-s001.pdf]

ONLINE SUPPLEMENT

The role of skin inflammation, barrier dysfunction, and oral tolerance in skin sensitization to gluten-derived hydrolysates in a rat model

Jeppe Madura Larsen[1], Anne-Sofie Ravn Ballegaard[1], Angela Serrano Dominguez[1], Nanna Jordahn Kristoffersen[1], Natalia Zofia Maryniak[1], Arielle Vallee Locke[1], Sahar Kazemi[2], Michelle Epstein[2], Charlotte Bernhard Madsen[1], Katrine Lindholm Bøgh[1]

[1]National Food Institute, Technical University of Denmark, Kgs. Lyngby, Denmark.

[2]Department of Dermatology, Medical University of Vienna, Vienna, Austria.

SUPPLEMENTARY FIGURES

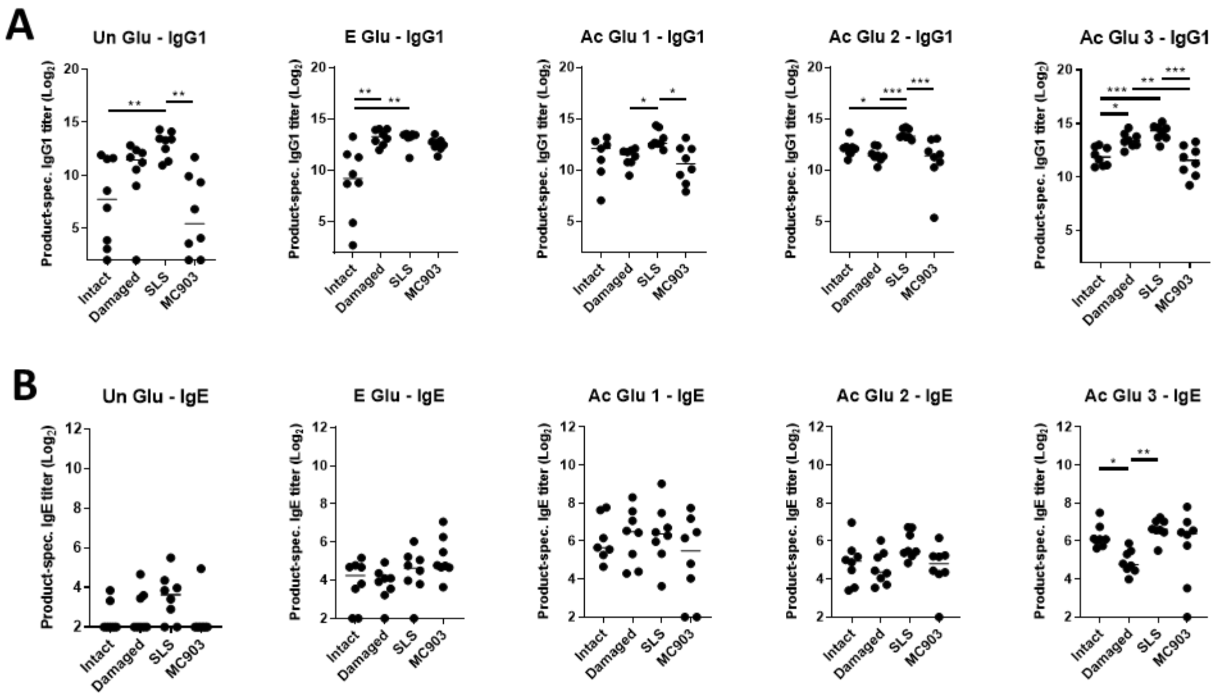

FIGURE S1 - Product-specific IgG1 and IgE levels following application of gluten-derived products to intact skin, barrier disrupted skin, or inflamed skin induced by SLS or MC903 in wheat-naïve rats. Product-specific (**A**) IgG1 and (**B**) IgE levels following 5 weeks of application (Day 42) of unmodified gluten (Un Glu), enzyme hydrolyzed gluten (E Glu), or acid hydrolyzed gluten (Ac Glu 1-3) products to intact skin, barrier disrupted skin (damaged), or inflamed skin induced by SLS or MC903 in wheat-naïve Brown Norway rats. Each symbol represents a single rat and horizontal lines indicate median values (n = 7 - 8 per group). The level of statistically significant differences between indicated groups are shown using asterisks: *p < 0.05. **p < 0.01. ***p < 0.001.

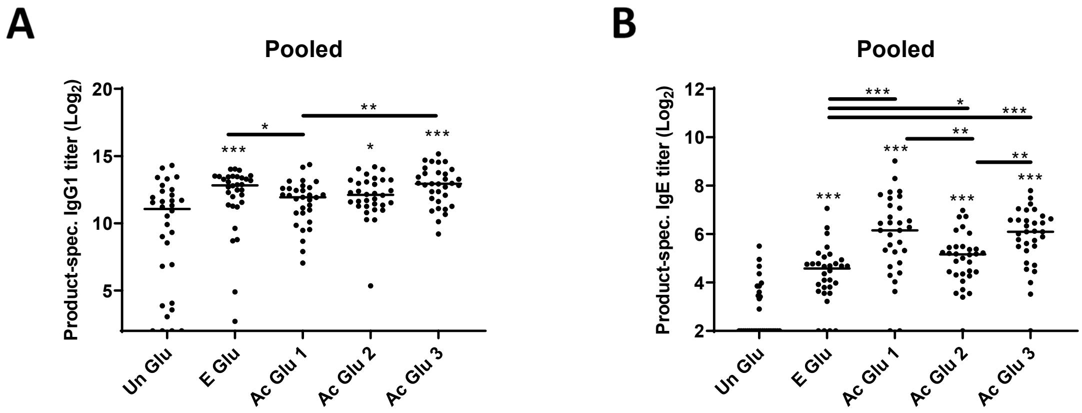

FIGURE S2 - Product-specific IgG1 and IgE levels following application of gluten-derived products to intact skin, barrier disrupted skin, or inflamed skin induced by SLS or MC903 in wheat-naïve rats. Product-specific (**A**) IgG1 and (**B**) IgE levels following 5 weeks of application (Day 42) of unmodified gluten (Un Glu), enzyme hydrolyzed gluten (E Glu), or acid hydrolyzed gluten (Ac Glu 1-3) products to intact skin, barrier disrupted skin (damaged), or inflamed skin induced by SLS or MC903 in wheat-naïve Brown Norway rats. Data shown are pool from the four different skin conditions. Each symbol represents a single rat and horizontal lines indicate median values (n = 31 - 32 per group). Statistically significant differences between groups are indicated as: *p < 0.05. **p < 0.01. ***p < 0.001.

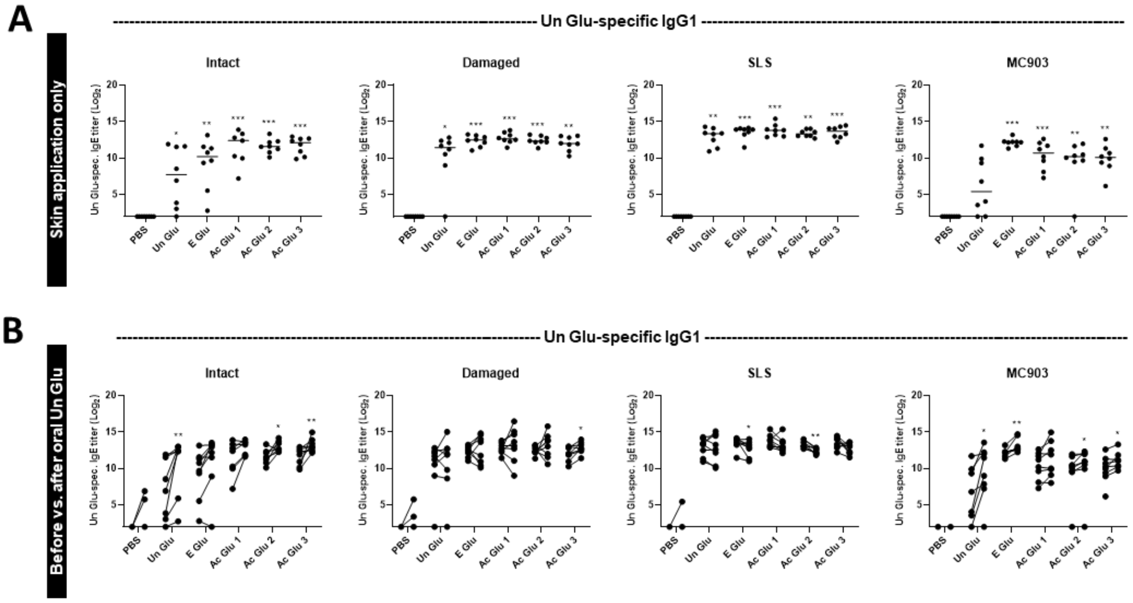

FIGURE S3 - Cross-reactivity to unmodified gluten following application of gluten-derived products to intact skin, barrier disrupted skin, or inflamed skin induced by SLS or MC903 in wheat-naïve rats. Unmodified gluten (Un Glu)-specific IgG1 levels (**A**) before (Day 42) and (**B**) before (Day 42) vs. after (Day 57) two oral gavages of Un Glu following 5 weeks of application of Un Glu, enzyme hydrolyzed gluten (E Glu), or acid hydrolyzed gluten (Ac Glu 1-3) products to intact skin, barrier disrupted skin (damaged), or inflamed skin induced by SLS or MC903 in wheat-naïve Brown Norway rats. Each symbol represents a single rat and horizontal lines indicate median values (n = 7 - 8 per group). Statistically significant differences between groups are indicated as: *p < 0.05. **p < 0.01. ***p < 0.001.

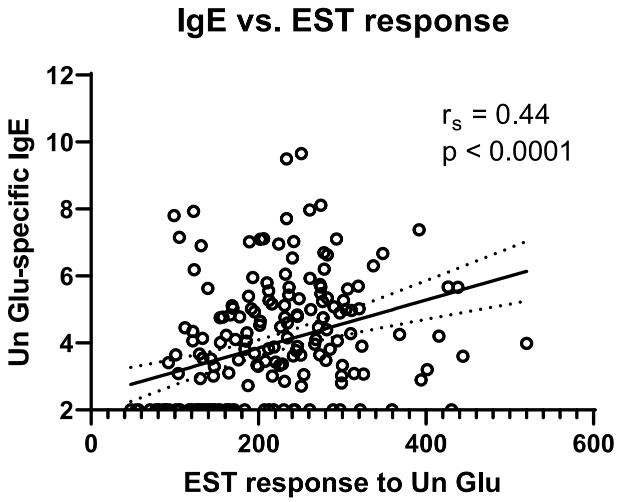

FIGURE S4 – Correlation between unmodified gluten-specific IgE levels and Ear Swelling Test responses to unmodified gluten after skin application of gluten-derived products and subsequent oral administration of unmodified gluten in wheat-naïve Brown Norway rats. Unmodified gluten (Un Glu)-specific IgE levels in serum after two oral gavages of Un Glu following 5 weeks of application of Un Glu, enzyme hydrolyzed gluten (E Glu), or acid hydrolyzed gluten (Ac Glu 1-3) products to intact skin, barrier disrupted skin (damaged), or inflamed skin induced by SLS or MC903 in wheat-naïve Brown Norway rats (Day 57). Ear Swelling Test (EST) response to intradermal Un Glu injections at Day 56. Each symbol represents a single rat (n = 190). Correlation was analyzed by Spearman's rank correlation test. Line shows linear regression with 95% confidence bands.
